# Acetyl L-Carnitine Nanoparticles Modulate Neuronal and Inflammatory Responses in In Vitro Cell Model

**DOI:** 10.3390/ijms27157024

**Published:** 2026-08-05

**Authors:** Alessia Mariano, Benedetta Brugnoli, Iolanda Francolini, Sergio Ammendola, Anna Scotto d’Abusco

**Affiliations:** 1 Department of Biochemical Sciences, Sapienza University of Rome, P.le Aldo Moro 5, 00185 Roma, Italy; alessia.mariano@uniroma1.it; 2 BeSSA Department of Wellbeing, Health and Environmental Sustainability, Sapienza University of Rome, 02100 Rieti, Italy; 3 Department of Chemistry, Sapienza University of Rome, P.le Aldo Moro 5, 00185 Rome, Italy; benedetta.brugnoli@uniroma1.it (B.B.); iolanda.francolini@uniroma1.it (I.F.); 4 Ambiotec di Sergio Ammendola, Via Appia Nord 47, 04012 Cisterna di Latina, Italy; ammendola@ambiotec.it

**Keywords:** nanoparticles, neuroprotection, acetyl L-carnitine, S100B, nerve growth factor, NF-κB pathway, drug delivery

## Abstract

Acetyl L-carnitine is an ester of the trimethylated amino acid L-carnitine with well-documented neuroprotective properties. Despite its ability to cross the blood–brain barrier, acetyl L-carnitine requires high and repeated doses to achieve and maintain therapeutic concentrations in the central nervous system. To overcome these limitations, nanotechnology-based delivery systems have emerged as a promising strategy to improve drug bioavailability, targeting, and therapeutic efficacy. In this study, we evaluated the efficacy of nanoparticle-based formulations of acetyl L-carnitine in comparison with its conventional bulk form using in vitro cultures of SH-SY5Y neuroblastoma cell line. The ALC nanoparticles were produced through an organic solvent-free mechanical ball milling process employing a planetary ball mill. The dimension and stability of the nanoparticles were analyzed by Dynamic Light Scattering and Thermogravimetric Analysis. The biological effects were evaluated using quantitative Real Time-Polymerase Chain Reaction, Enzyme-linked Immunosorbent Assay and immunofluorescence experiments. ALC nanoparticles, at low concentration of nanoparticles compared to the non-nanoparticle form, were able to decrease the alarmin S100B release, pro-inflammatory interleukin mRNA and protein expression as well as p65 activation, confirming their involvement in NF-κB pathway. Moreover, it was able to stimulate the nerve growth factor release and to increase intracellular Ca^++^ levels, showing neuroprotective effects in addition to anti-inflammatory ones. Our findings allow us to highlight the therapeutic potential of ALC nanoparticles for neurological disorders, with the prospect of enhancing efficacy while reducing dosage and administration frequency.

## 1. Introduction

Acetyl L-carnitine (ALC) is an ester of L-carnitine synthesized in the human brain, liver, and kidneys by the enzyme ALC-transferase. ALC facilitates the transport of acetyl-CoA into mitochondria during fatty acid oxidation, enhances acetylcholine production, and stimulates the synthesis of proteins and membrane phospholipids. It has been investigated in several experimental and clinical studies as an adjuvant treatment in patients with dementia, aging-related cognitive decline, Alzheimer’s disease, multiple sclerosis–related fatigue, autism spectrum disorders, peripheral neuropathies, and other common neurological conditions [1,2,3]. At the cellular level, ALC exhibits multiple properties that converge into neuroprotective effects. It donates its acetyl group for the synthesis of acetylcholine and other neurotransmitters, exerts anti-inflammatory and antioxidant activity, stimulates the release of nerve growth factor (NGF), and enhances metabolic and cholinergic responses [4,5]. Both L-carnitine and ALC can be administered orally, intravenously, or intramuscularly. They are absorbed in the intestine by simple diffusion and transported into tissues via active transport mechanisms [6]. At the level of the blood–brain barrier (BBB), ALC crosses more readily than L-carnitine and is rapidly metabolized. Although ALC is able to reach the brain, therapy with this supplement requires repeated administrations and relatively high doses to maintain stable therapeutic levels [7]. Considering that ALC is often prescribed as an adjuvant therapy in patients with dementia, cognitive impairment, and other neurological disorders, frequent dosing may reduce patient compliance and adherence to treatment. Therefore, the development of nanoparticle-based formulations of ALC may represent a significant therapeutic advantage, potentially reducing the required dose and frequency of administration while maintaining or enhancing therapeutic efficacy in these patients.

In recent years, nanotechnology has been extensively used in medical fields both for the diagnosis and treatment of various diseases [8]. As a core branch of this discipline, nanomedicine exploits the nanoscale manipulation of matter to design innovative theranostic platforms, advanced imaging agents, and targeted formulations [9]. Nanoparticles (NPs) represent the main products of nanotechnology applied to medicine. Due to their unique physico-chemical properties, such as a high surface area-to-volume ratio and nanoscale dimensions, NPs offer several pharmacokinetic advantages over larger particles [10]. Depending on the type of nanomaterial, NPs exhibit enhanced tissue and cellular absorption and accumulation, as well as targeted action on specific cells, enabling selective drug release. This results in increased bioavailability and reduced systemic toxicity [11]. To fully exploit these medical advantages, the choice of the NP synthesis method is critical, as it directly dictates particle size, morphology, and surface chemistry. Generally, NP synthesis strategies are divided into “bottom-up” chemical/biological approaches and “top-down” physical methods [12]. However, conventional chemical routes often rely on toxic organic solvents, high temperatures, and complex purification steps, which limit their scalability and clinical translation.

To overcome these limitations, mechanochemically driven synthesis has emerged as a powerful alternative for medical-grade NPs. This strategy induces physico-chemical transformations through mechanical forces (such as milling, grinding, or shearing) without the need for organic solvents [13]. This organic solvent-free nature drastically reduces environmental toxicity and eliminates hazardous chemical residues in the final product, a feature of paramount importance for biocompatibility and regulatory approval. Furthermore, mechanochemical synthesis offers high reproducibility, scalability, exceptional energy efficiency, and the unique ability to induce polymorphic transitions or form novel co-crystals that cannot be achieved via traditional wet chemistry. Consequently, this sustainable and versatile method ensures the production of highly pure, biocompatible NPs with tailored properties optimized for advanced drug delivery and therapeutic applications.

Given these pharmacokinetic characteristics, numerous studies in recent years have focused on the use of nanoparticle-based formulations to deliver drugs across the BBB for the treatment of brain disorders and increase bioavailability [14]. The BBB is a physical barrier formed by endothelial cells connected by continuous, non-fenestrated tight junctions, which regulate the supply of essential nutrients to the brain and prevent the entry of potentially harmful substances that could compromise its integrity. Due to these characteristics, most drugs are unable to cross the BBB and reach their target site within the central nervous system (CNS) [15,16].

The aim of this study was to evaluate the efficacy of nanoparticle formulations of ALC (ALC NP) in in vitro cultures of SH-SY5Y nervous cells compared to non-nanoparticle ALC (commercial formulation in bulk form). The studied nanoparticles were obtained through a top–down ball milling approach. Specifically, a planetary ball mill reduces the size of the bulk powder to obtain nanoparticles through the mechanical impact of small beads of inert material [17,18,19]. Within this framework, the experimental focus was directed toward investigating the biological safety, anti-inflammatory and neuroprotective potential of these nano-formulations, thereby establishing a fundamental baseline for future translational applications.

## 2. Results

### 2.1. Preparation and Physical Characterization of Acetyl L-Carnitine Nanoparticles

**Figure 1:**
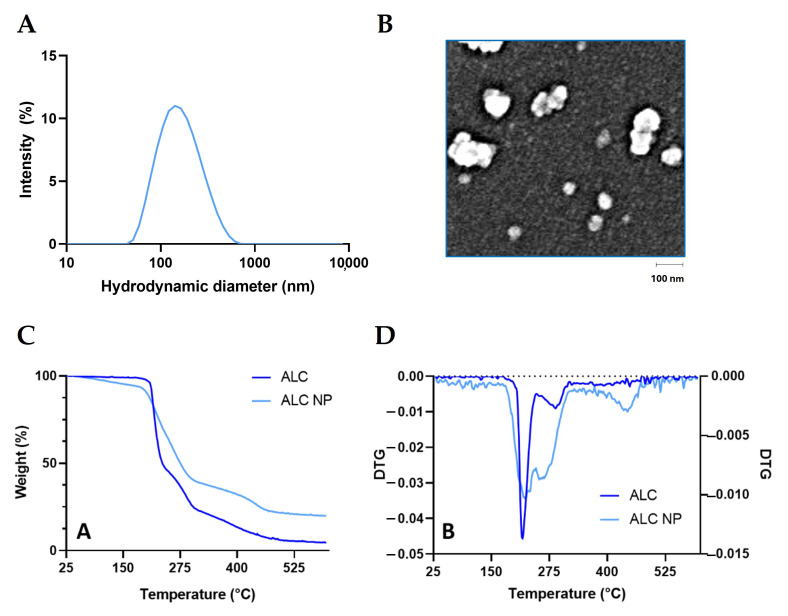
ALC and ALC NP characterization. (**A**) Size distribution of ALC NP. (**B**) SEM analysis of ALC NP. (**C**) Thermogravimetric and (**D**) derivative (DTG) curves of ALC and ALC NP.

ALC NP was prepared through a top–down methodology starting from a mixture of ALC bulk and raw polyvinylpyrrolidone (PVP) powders. The produced nanoparticles exhibit a high-purity composition consisting of active drug, ALC, and a low amount of polymeric additive, PVP, in a mass ratio ALC:PVP = 50:1. Since PVP is present in very low amounts, it acts strictly as an interfacial stabilizer rather than a structural matrix shell. The size and polydispersity of ALC NP were determined using DLS. The resulting size distribution curve, the hydrodynamic diameter, and the PDI value are summarized in Figure 1A and Figure S1. ALC NP displayed a monomodal size distribution with a mean hydrodynamic diameter in a range of 135–155 nm and PDI = 0.2 and a zeta-potential of 4.89 mV. Moreover, Scanning Electron Microscope (SEM) analysis showed the presence of the nanoparticles with spherical shape and aggregates with irregular spherical shape (Figure 1B). In all the experiments described below, a preparation with a hydrodynamic diameter of 142 nm was used. PVP alone both in bulk and NP form was analyzed by DLS, finding that the PVP in bulk form had a hydrodynamic diameter with a highly polydisperse, bimodal distribution, whereas PVP NP showed a very small hydrodynamic diameter with a well-defined size centered between 15 and 20 nm (Figure S2 and Table S1). Thus, the hydrodynamic diameter of ALC NP measured by DLS analysis undoubtedly represents the hydrodynamic diameter of the ALC NP complex (ALC:PVP = 50:1).

To assess ALC and ALC NP thermal stability before and after formulation with PVP, TGA was performed (Figure 1C,D). The ALC thermogram exhibited a sharp and non-concerted two-step degradation profile, showing a first weight loss at approximately 215 °C. Stoichiometric calculations confirmed that this primary weight loss of 50.26% corresponds to the simultaneous decomposition of both the acetyl group (21.18% theoretical) and the quaternary ammonium moiety (29.08% theoretical), which together account for 50.26% of the total molar mass. The remaining mass loss at 290 °C (49.8%) is associated with the ALC scaffold. The ALC NP degradation profile was gradual and concerted in a similar temperature range (215–260 °C) (Figure 1D, light blue curve). Additionally, a peak at 440 °C confirmed the presence of the PVP, while the 22% residual mass is attributed to the formation of stable carbonaceous char.

**Figure 2:**
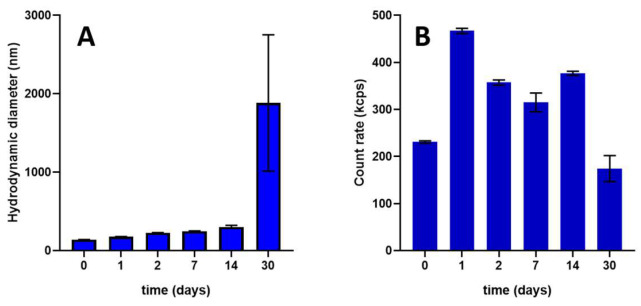
Time stability of ALC NP in water by monitoring hydrodynamic diameter (**A**) and count rate (**B**).

Moreover, ALC NP stability was evaluated by monitoring the hydrodynamic diameter and count rate over time in water, by always analyzing the same suspension (Figure 2 and Table 1). As illustrated in the bar graph, a significant increase in both size and PDI was observed over a 30-day period, by the end of which the highest values, approximately 1900 nm and PDI = 0.7, were reached. In contrast, the count rate remained almost constant for up to two weeks, after which it sharply decreased. The reduction in count rate, together with the pronounced increase in particle size, is consistent with the formation of larger aggregates and possible sedimentation phenomena, which reduce the number of particles effectively detected in suspension.

### 2.2. ALC and ALC NP Effects on SH-SY5Y Cell Line Viability

**Figure 3:**
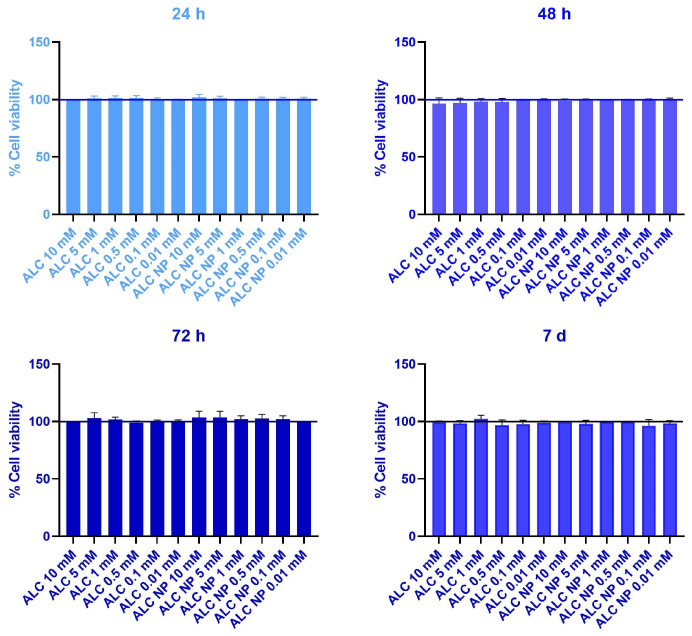
Cell viability was assessed by the MTS colorimetric method, and SH-SY5Y were treated with six concentrations from 10 mM to 0.01 mM of ALC and ALC NP, for 24, 48, 72 h and 7 days. Cell viability of treated samples was normalized to the untreated cells (CTLs) represented by horizontal lines.

SH-SY5Y cell line was chosen as a model of nervous cells to study in vitro the effects of acetyl L-carnitine, in both bulk and nanoparticle form, on the central nervous system. In order to evaluate their biocompatibility, ALC and ALC NP freshly prepared solution containing nanoparticles of 142 nm in size were added to the SH-SY5Y neuroblastoma cells to evaluate their effects on cell viability at different concentrations (10 mM, 5 mM, 1 mM, 0.5 mM, 0.1 mM and 0.01 mM) for up to 7 days of treatment. The MTS assay results showed ALC, in both bulk and nanoparticle form, did not exert any detrimental effects under our experimental conditions (Figure 3). Further experiments were performed using the two lowest and intermediate non-cytotoxic concentrations, 0.01 mM and 0.1 mM, always using a freshly prepared solution containing nanoparticles of 142 nm in size, in order to maintain a focus on dose minimization, in line with potential therapeutic translation strategies.

### 2.3. Effects of ALC and ALC NP on S100B Secretion

**Figure 4:**
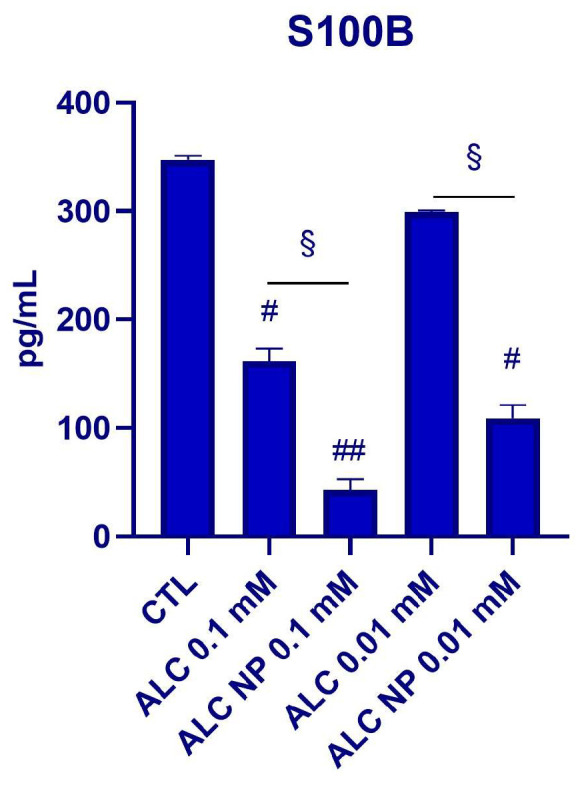
Effects of ALC and ALC NP on S100B expression level. Cells were left untreated (CTL) or treated with 100 μM and 10 μM concentrations of ALC or ALC NP for 24 h. After treatments, the supernatants were collected and analyzed by ELISA. The results are reported as pg/mL and are expressed as mean ± Standard Deviation (SD) of data obtained by three independent experiments. # *p* < 0.05 ALC or ALC NP vs. CTL, ## *p* < 0.01 ALC NP vs. CTL, § *p* < 0.05 ALC NP vs. ALC.

Cerebral aging is associated with many structural, biochemical, and molecular changes in the brain that are responsible for tissue atrophy, inflammation and oxidative stress at the cellular level, and impaired neurotransmitter release. Overall, these alterations contribute to progressive cognitive decline. The S100B protein is an alarmin able to activate inflammatory pathways, such as the NF-κB complex involved in cytokine release. Recently, its action at the central nervous tissue level has been assessed [20,21]. With the aim of evaluating the effects of acetyl L-carnitine on the S100B secretion, SH-SY5Y cells were treated with ALC and ALC NP at concentrations of 0.1 mM and 0.01 mM. The results showed that ALC was able to decrease the S100B secretion only at 0.1 mM concentration, whereas ALC NP statistically decreased this marker at both analyzed concentrations, although the 0.1 mM was more effective than 0.01 mM (Figure 4).

### 2.4. Effects of ALC and ALC NP on NF-κB Activation

**Figure 5:**
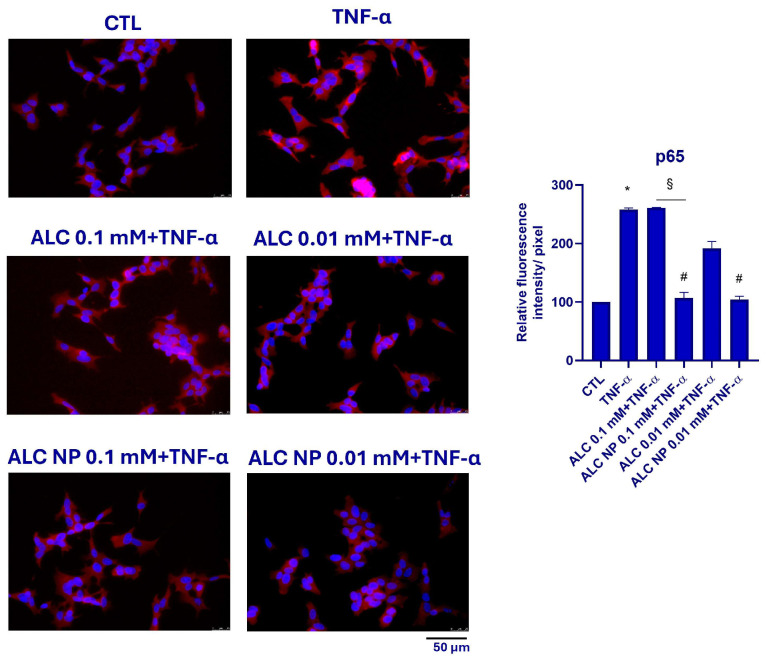
Effects of ALC and ALC NP on p65 production. Cells were treated with 100 μM and 10 μM ALC or ALC NP for 1 h and then stimulated with 10 ng/mL TNF-α for 10 min and then analyzed by immunofluorescence using anti-p65 primary antibodies and Alexa Fluor 594 (red) secondary antibody. Nuclei were stained with DAPI (original magnification 40×). The bar graph represents the pixel intensities in the region of interest, obtained by ImageJ. * *p* < 0.05 TNF-α vs. CTL; # *p* < 0.05 ALC NP + TNF-α vs. TNF-α, § *p* < 0.05 ALC NP 0.1 mM + TNF-α vs. ALC 0.1 mM + TNF-α.

**Figure 6:**
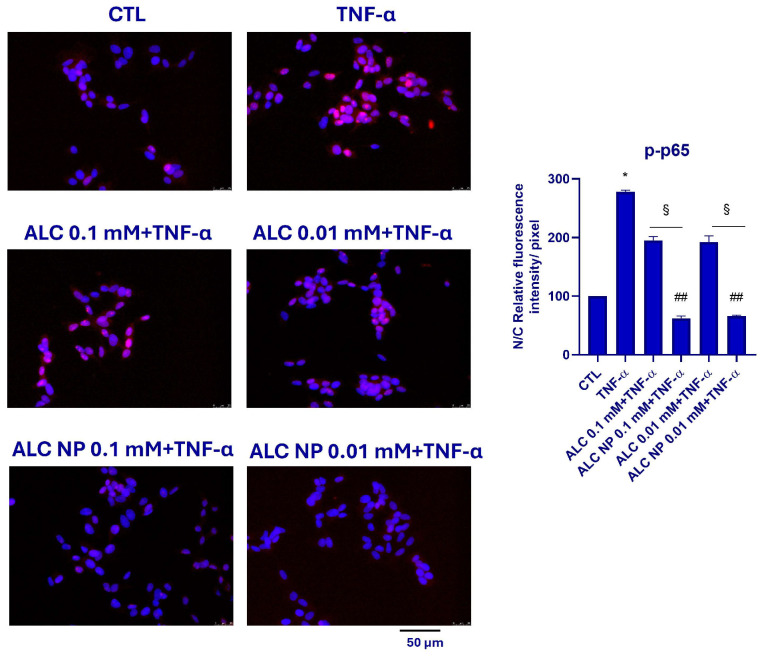
Effects of ALC and ALC NP on p65 phosphorylation (p-p65). Cells were treated with 100 μM and 10 μM ALC or ALC NP for 1 h and then stimulated with 10 ng/mL TNF-α for 10 min and then analyzed by immunofluorescence using anti-p-p65 primary antibody and Alexa Fluor 594 (red) secondary antibody. Nuclei were stained with DAPI (original magnification 40×). The bar graph represents the pixel intensities in the region of interest, obtained by ImageJ, applying the formula *N/C = Fnucl/Fcyt*. * *p* < 0.05 TNF-α vs. CTL; ## *p* < 0.01 ALC NP + TNF-α vs. TNF-α, § *p* < 0.05; ALC NP + TNF-α vs. ALC + TNF-α.

NF-κB is a family of transcription factors, which includes several isoforms, such as p65. This protein is present in the cytoplasm of cells in an inactive form, inhibited by nuclear factor of κ light polypeptide gene enhancer in B-cell Inhibitor α (IκBα); after pro-inflammatory stimulation, p65 is phosphorylated (p-p65) and then translocated into the nucleus to activate transcription. Considering the effects of S100B in stimulating the activation of NF-κB, the effects of ALC and ALC NP were evaluated on the activation of p65. To mimic the pro-inflammatory environment of an aged brain, SH-SY5Y cells were stimulated with 10 ng/mL TNF-α and treated with 0.1 mM and 0.01 mM ALC and ALC NP. TNF-α stimulated the production of p65, which was decreased at the control level by both analyzed concentrations of ALC NP, whereas ALC in bulk form was not effective (Figure 5). Notably, ALC NP were able to inhibit the p65 phosphorylation and, in turn, its nuclear migration. ALC in bulk form was only slightly able to inhibit p65 activation, which was not statistically significant. As shown in Figure 6, p-p65 nuclear translocation was prominent in TNF-α stimulated cells, whereas it was almost completely absent in cells stimulated with TNF-α and treated with ALC NP.

### 2.5. Effects of ALC and ALC NP on Interleukins Production

**Figure 7:**
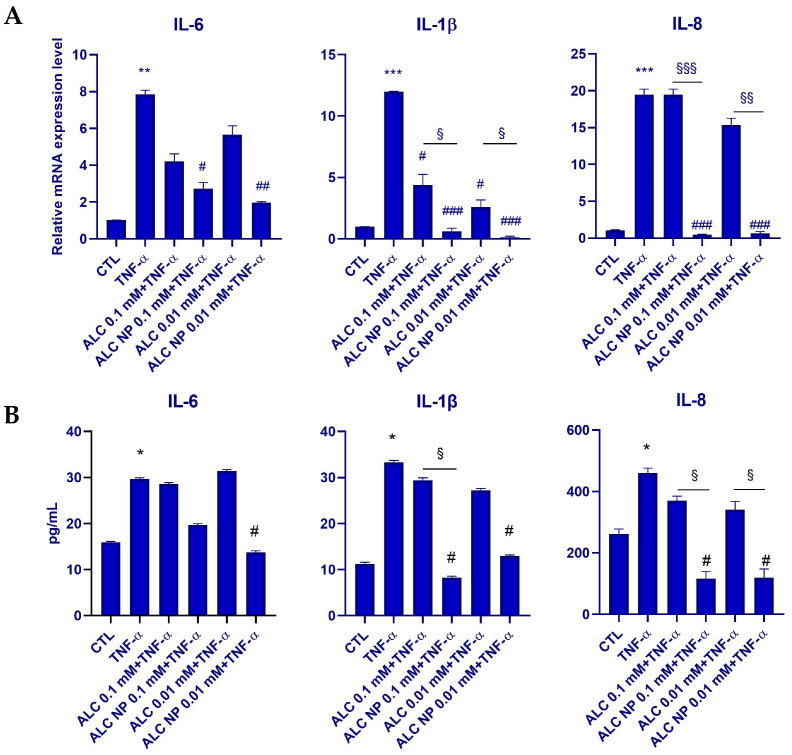
Effects of ALC and ALC NP on interleukins expression levels under TNF-α stimulus. (**A**). Cells were left untreated (CTL), or stimulated with 10 ng/mL TNF-α for 30 min, or treated with 100 μM and 10 μM concentrations of ALC or ALC NP for 1 h and then stimulated with 10 ng/mL TNF-α for 30 min. After treatments, mRNA was extracted and analyzed by RT-PCR. IL-6, IL-1β and IL-8 mRNA levels were reported as relative mRNA expression levels with respect to 18S mRNA (2^-ΔΔCt^ method). (**B**). Cells were left untreated (CTL) or stimulated with 10 ng/mL TNF-α for 1 h or treated with 100 μM and 10 μM concentrations of ALC or ALC NP for 1 h and then stimulated with 10 ng/mL TNF-α for 1 h. After treatments the supernatants were collected and analyzed by ELISA. The results are reported as pg/mL and are expressed as mean ± SD of data obtained by three independent experiments. * *p* < 0.05, ** *p* < 0.01 and *** *p* < 0.005 TNF-α vs. CTL; # *p* < 0.05, ## *p* < 0.01 and ### *p* < 0.005 ALC + TNF-α or ALC NP + TNF-α vs. TNF-α, § *p* < 0.05, §§ *p* < 0.01 and §§§ *p* < 0.005 ALC NP + TNF-α vs. ALC + TNF-α.

Among the consequences of NF-κB activation, cells produce pro-inflammatory interleukins. To evaluate the effects of acetyl L-carnitine in bulk and nanoparticle form, SH-SY5Y cells were stimulated with 10 ng/mL TNF-α and treated with 0.1 mM and 0.01 mM of ALC and ALC NP. Our analysis showed that mRNA levels of pro-inflammatory cytokines IL-6 and IL-1β were decreased by both ALC and ALC NP, even if the decrease in IL-6 by ALC was not statistically significant. Regarding the chemokine IL-8, only ALC NP was able to decrease its mRNA level. However, ALC NP proved to be more effective than the bulk form in counteracting the pro-inflammatory stimulus induced by TNF-α; in particular, ALC NP was able to reduce the expression of IL-1β and IL-8 below control levels (Figure 7A).

Also at the protein level, ALC NP resulted more effective than bulk ALC in reducing the interleukin secretion in the culture medium. Specifically, the nanoparticle formulation was statistically effective in reducing the pro-inflammatory markers, even if the decrease due to the 0.1 mM concentration treatment was not statistically significant. The bulk form was not able to modulate the interleukin secretion (Figure 7B). Moreover, to ensure that PVP exerted no biological effects on the cells, SH-SY5Y cells were treated with PVP alone, both in bulk and nanoparticle form, finding that they were not able to modulate the mRNA expression of these pro-inflammatory cytokines (Figure S3).

### 2.6. Effects of ALC and ALC NP on Nerve Growth Factor Production

**Figure 8:**
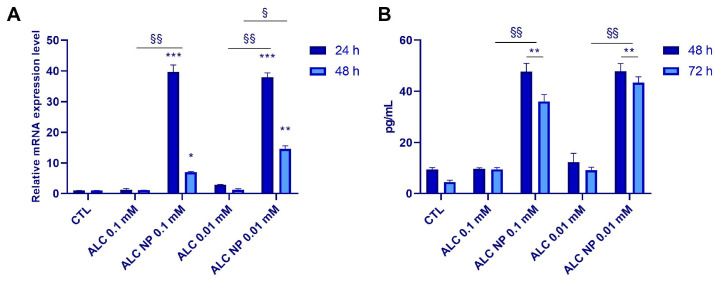
Effects of ALC and ALC NP on NGF/NGFβ production level. (**A**) Cells were left untreated (CTL) or treated with 100 μM and 10 μM concentrations of ALC or ALC NP for 24 h, 48 h and 72 h. A. After 24 h and 48 h-treatment cells were harvested, and mRNA was extracted and analyzed by RT-PCR. NGF mRNA level was reported as relative mRNA expression level with respect to 18S mRNA (2^−ΔΔCt^ method). (**B**). After 48 h and 72 h of treatment, the supernatants were collected, and analyzed by ELISA; the results are reported as pg/mL. The mRNA and protein levels are expressed as mean ± SD of data obtained by three independent experiments. * *p* < 0.05 ALC NP 0.1 mM (48 h) vs. CTL, ** *p* < 0.01 ALC NP (48 h) vs. CTL, *** *p* < 0.005 ALC NP (24 h) vs. CTL, § *p* < 0.05 and §§ *p* < 0.01 ALC NP vs. ALC.

Nerve growth factor (NGF) is a neurotrophic protein necessary for normal development and function of the mammalian nervous system. It is involved in brain cholinergic function, and its production is reduced in aging human CNS leading to brain neuronal damage, loss of synaptic plasticity, and cognitive decline. To this end, the effects of ALC NP on NGF expression and release were studied. SH-SY5Y cells were treated with 0.1 mM and 0.01 mM ALC and ALC NP for 24 h, 48 h and 72 h, revealing that ALC did not increase the expression and secretion of NGF at any of the concentrations or time points analyzed (Figure 8A,B). In contrast, ALC NP was able to induce the expression and release of NGF at both concentrations and at all tested time points (Figure 8A,B). The NGF mRNA modulation was higher at 24 h than 48 h, whereas the protein release at 48 h and 72 h was comparable.

### 2.7. Effects of ALC and ALC NP on Cytoplasmic Ca^++^ Level

**Figure 9:**
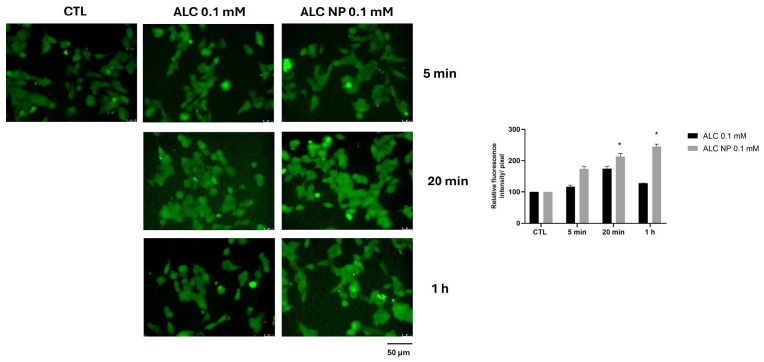
Effects of ALC and ALC NP on intracellular Ca^++^ concentration. Cells were treated with 100 μM ALC or ALC NP for 5 min, 20 min and 1 h, then the amount of intracellular Ca^++^ concentration was measured by Calcium Green-1 AM. The densitometric analysis (right) was performed by ImageJ. Results are expressed as mean ± SD of data obtained by three independent experiments. * *p* < 0.05 ALC NP vs. CTL.

Calcium is a second messenger involved in several cell functions, such as cell growth, differentiation, apoptosis and nervous transmission. To evaluate the effects of ALC and ALC NP on cytoplasmic modulation of calcium ions, cells were treated with both formulations for 5 min, 20 min and 1 h at 0.01 mM and 0.1 mM concentrations. The 0.01 mM concentration of ALC and ALC NP stimulated intracellular Ca^++^, although not in a statistically significant manner (Figure S4), whereas the 0.1 mM concentration of ALC NP was able to increase intracellular calcium levels in a time-dependent way, with the maximum effect observed after 1 h of treatment (Figure 9). ALC in bulk form, at the same concentration, showed a non-statistical increase only after a 20 min treatment (Figure 9).

## 3. Discussion

Aging is an irreversible process associated with changes in several molecular and cellular pathways, among these genomic instability, telomere attrition, epigenetic alterations as well as mitochondrial dysfunction, cellular senescence, deregulated nutrient sensing and chronic inflammation [22]. With the progression of aging, all these alterations become cumulative, leading to the functional decline of the whole organism. Those tissues composed mainly of postmitotic cells, such as the brain, are particularly sensitive to the aging effects, due to the limited ability to renew themselves [23]. On the other hand, terminally differentiated neurons are considered fundamental for the maintenance of genetic information [23]. Accumulation of aging alterations in the nervous systems contributes to tissue dysfunction and several age-related disorders [24].

Inflammation is a main protective event against several types of insult, and with aging, several kinds of inflammation can be observed, as low-grade, controlled, asymptomatic, chronic and systemic states [25]. While low-grade inflammation, lasting for a short period, could have beneficial effects, uncontrolled and prolonged inflammation can result in chronic neurodegenerative disorders, such as Alzheimer’s and Parkinson’s diseases [26]. Diet, nutraceutical supplementation, and regular exercise could help in limiting the neuronal senescence and thus the onset of neurodegenerative diseases.

Acetyl L-carnitine (ALC) oral administration is considered a good strategy to limit neurodegeneration, due to its ability to cross the blood–brain barrier (BBB) [27]. Previous studies suggest that ALC possesses intrinsic BBB permeability mediated, at least in part, by the organic cation/carnitine transporter (OCTN)2. In particular, Kido et al. demonstrated the functional involvement of OCTN2 in the transport of ALC across the BBB using both in vivo and in vitro models [28], while Inano et al. further confirmed OCTN2-mediated ALC permeation in mouse brain microdialysis studies [27]. Nevertheless, reducing the dosage is desirable to increase patient compliance.

Previously, ALC was successfully utilized as a surface-modifying ligand in liposomal formulations to enhance cellular uptake and protect the carrier system during delivery [29]. While this targeting strategy significantly improves delivery efficiency and cell internalization, ligand-functionalized liposomes typically involve a high lipid-to-ligand ratio. Consequently, the large amount of lipid excipients relative to the active payload remains an important consideration during preparation. In the present study, ALC NP were prepared using PVP as stabilizer, using a top–down method. The analysis of the produced nanoparticles showed that PVP represents a good strategy to obtain stable and small-sized formulation without excessive excipients and solvents. DLS analysis revealed that the ALC NP displayed a monomodal size distribution, characterized by a mean hydrodynamic diameter in a range of 135–155 nm and a PDI of 0.2. To accurately represent the physical dimensions of the sample and account for the intensity-weighting effects of larger trace aggregates (I ∝ d6), the number-weighted distribution was also reported in Supplementary Materials. The distribution by number profile exhibited a narrower population peak centered at approximately 70 nm, which completely terminates below 250 nm.

The thermogravimetric curve of pure ALC exhibited a characteristic two-step degradation, with an initial mass loss at approximately 215 °C (acetyl and ammonium quaternary groups simultaneous decomposition) and a subsequent stage at 290 °C (ALC backbone). On the other hand, the degradation profile of ALC NP showed a concerted process within the same temperature range of pure ALC. This thermal transition from a sharp, multi-step drop to a concerted thermal event suggests that the PVP matrix modulates the thermal degradation kinetics. Although the physical state of the drug in the NP was not investigated, the observed thermal behavior may indicate a reduction in drug crystallinity. Consistent with this hypothesis, the preparation method employed in this study, ball milling, is known to induce drug amorphization through the mechanical stress imparted during the milling process [30,31]. This thermal behavior is consistent with reports on L-carnitine (LC) functionalized nanocomposites, where surface modification leads to distinct organic decomposition phases up to 500 °C [32,33]. Additionally, the presence of PVP in the current study was confirmed by a degradation peak at 440 °C. This aligns with literature values for pure PVP, which typically shows major thermal decomposition between 350 °C and 475 °C due to the breakdown of the carbonic backbone and pyrrolidone groups [34]. Furthermore, the 22% residual mass observed at the end of the thermogravimetric curve is attributed to the formation of a stable carbonaceous char; as PVP undergoes simultaneous main-chain radical cleavage and β-elimination, these highly reactive radical intermediates can undergo secondary inter-chain cross-linking, resulting in a carbon residue that remains stable under inert atmosphere [35,36].

The long-term stability of the dissolved ALC NP was assessed by monitoring the hydrodynamic diameter and the count rate over time. As observed, both parameters increased significantly over a 30-day period, ultimately reaching a maximum diameter of approximately 1900 nm and a PDI of 0.7. The count rate data show an initial increase in kcps, which is consistent with the formation of larger aggregates. Subsequently, the count rate remains relatively stable for up to two weeks before decreasing on day 30. This decrease, together with the marked increase in particle size observed at the same time point, is consistent with aggregation and partial sedimentation phenomena, which reduce the number of particles effectively detected in suspension. These results suggest that while the addition of PVP initially stabilizes the top–down milled nanoparticles by acting as a surface-dispersing agent to prevent immediate post-milling re-aggregation [37,38], its protective interfacial effect diminishes over time [39]. Since PVP is present in very low amounts (mass ratio ALC:PVP = 50:1), acting strictly as an interfacial stabilizer rather than a structural matrix shell, the subsequent increase in particle size indicates a physical evolution of the suspension. This behavior is likely driven by the partial desorption, displacement, or structural rearrangement of the polymer chains at the nanoparticle–water interface over prolonged storage, which progressively compromises the initial repulsive barrier and leads to controlled aggregation [40,41]. Regarding surface charge, the slightly positive zeta-potential, 4.9 mV, confirms the lack of strong electrostatic repulsion, explaining the long-term aggregation tendency in aqueous media. For these reasons, ALC NPs were freshly dissolved before each cell-based experiment.

The successful miniaturization of ALC powder particle size could be considered optimal for nanoparticle-mediated drug delivery to the CNS, since particles in this dimensional range efficiently cross the BBB. Furthermore, the high surface-area-to-volume ratio enhances drug dissolution kinetics and facilitates interaction with cell membranes [42]. The low positive charge, measured by z-potential, could prevent acute cytotoxicity, hemolysis, and rapid opsonization followed by reticuloendothelial system clearance, typical of highly positively charged nanoparticles [43]. Simultaneously, this slight positive charge promotes favorable, gentle electrostatic interactions with the negatively charged phospholipid bilayers and sialic acid residues of brain endothelial membranes [44]. Importantly, because ALC NPs do not rely solely on passive charge-mediated uptake, this mild charge profile preserves the accessibility of the ALC moiety to interact with its specific transporter, OCTN2 [45]. For these reasons, the biological effects have been evaluated using the undifferentiated SH-SY5Y neuroblastoma cell line. This neuroblastoma cell line is usually used as a model for neurodegenerative diseases and neuroinflammation for in vitro studies, considering that they offer easier manipulation than primary neurons, but are still ideal for studying basic mechanisms [46].

S100B, a small Ca^++^-binding protein, has been described to have a double role on neurons: a neurotrophic role when expressed at low concentrations, and neurotoxic role when expressed at high concentrations [47,48]. In the brain, S100B is secreted by astrocytes, even if it is expressed by several types of cells, among them neurons [49], and is one of the most abundant soluble protein in the brain [50]. When S100B reaches high extracellular concentrations, it acts as a pro-inflammatory cytokine and its action is mediated, among others, by the RAGE receptor, leading to the activation of the NF-κB pathway [51].

In our in vitro model, using the neuroblastoma cell line SH-SY5Y, we measured the amount of secreted S100B in both untreated and ALC-treated cells. The findings showed that SH-SY5Y cells were able to secrete S100B, as also reported by other studies [52,53] and that ALC NP decreased the S100B secretion at both analyzed concentrations, whereas ALC in bulk form was poorly effective. The observed S100B level was lower than that observed in the cerebrospinal fluid of neurological patients, but our findings should be interpreted only as an in vitro modulation of basal S100B release rather than a direct model of neuroinflammation. Consequently, we analyzed the ability of ALC to inhibit the activation of p65, one of the transcription factors belonging to the NF-κB family, which is considered a master regulator of inflammatory response [54]. Activation of p65 consists of phosphorylation and subsequent nuclear translocation, where it is involved in gene transcription [55]. To simulate the inflammatory status, SH-SY5Y cells were stimulated with TNF-α after pre-treatment with ALC and ALC NP, demonstrating that ALC NP decreased the production of p65 and inhibited its activation by blocking both the phosphorylation and the nuclear migration at concentrations of both 0.1 mM and 0.01 mM, whereas ALC in bulk form at same concentrations was again not effective. Finally, we analyzed the production of pro-inflammatory interleukins, which are under the control of several transcription factors, including NF-κB [54]. Pro-inflammatory cytokines such as IL-6 and IL-1β are involved in the initiation of the immune response and are critical to counteract any injury [56]. We verified whether ALC could affect the production of IL-6 and IL-1β stimulated by TNF-α, finding that ALC NPs decreased the IL-6 production compared to that obtained with TNF-α stimulation, both at mRNA and protein level with greater effectiveness at the lower concentration of 0.01 mM. The decrease induced by ALC in bulk form was observed only at mRNA level and was not statistically significant. Regarding IL-1β, both ALC forms were effective in counteracting the TNF-α stimulation at mRNA level, but at protein level, once again, only ALC NP was effective. In CNS, pro-inflammatory chemokines are also implicated in cognitive impairment and neurodegeneration through several actions, such as the control of BBB permeability, regulation of neurogenesis, neuroprotection, and neurotoxicity [57,58]. IL-8, or CXCL8, is a pro-inflammatory chemokine involved in several disorders. Its main functions are the recruitment of immune system cells into the inflamed sites, whose cells among many other effects induce the impairment of BBB through the increase in metalloproteases [59]. In patients with neurodegenerative disorders, such as Alzheimer’s Disease, the IL-8 level was found to be increased [60]. Noteworthy, IL-8 secretion in low concentration can have neuroprotective effects [61]. In our model, the increase in IL-8 after stimulation with TNF-α was decreased, both at mRNA and protein level, only by ALC NP at both used concentrations. Thus, ALC NP proved to be effective in countering the inflammation typical of aging at a concentration of 0.01 mM which could be reached more easily with respect to the concentration usually obtained after ALC in bulk form.

In the aging brain, stimulating factors such as NGF are produced in very low amounts; in order to verify whether ALC NP could stimulate the production of NGF, we analyzed the SH-SY5Y cells with the same concentrations of ALC and ALC NP, finding that ALC NP at both concentrations stimulated NGF after 24, 48 and 72 h treatment, whereas ALC in bulk form was not effective. The mRNA expression was analyzed at 24 and 48 h, while the protein secretion evaluated at 48 and 72 h required longer times to be appreciated. Therefore, the efficacy of ALC NP to stimulate the NGF expression and secretion in SH-SY5Y neuroblastoma cell line is a novelty to the best of our knowledge, demonstrating that the cellular machinery for synthesizing and secreting NGF is present, but requires inductive input to become relevantly activated, as also reported by Borsani et al. [62].

Calcium is among the most ubiquitous second messengers in the nervous system signaling and it has a leading role in maintaining neuronal health and function [63]. Loss of intracellular calcium homeostasis may contribute to cognitive dysfunction [64]. SH-SY5Y cells treated with 0.1 mM ALC NP showed an increase in intracellular Ca^++^ at all analyzed times, even if the increase was statistically significant after 20 min and 1 h treatment, whereas the 0.1 mM ALC in bulk form was able to stimulate the increase in Ca^++^ only after 20 min treatment and in a non-statistically significant way. Cells treated with 0.01 mM ALC and ALC NP poorly stimulated the increase in Ca^++^ at all analyzed times.

NGF is crucial for differentiation and maintenance of neuronal cells through many well-characterized signaling pahtways, among which is the increase in intracellular Ca^++^, acting on several calcium channels localized on different cell types [65,66,67,68]. In agreement with this picture, we found that ALC NP is able to stimulate both the intracellular calcium concentration increase and NGF secretion. Considering that NGF cannot cross the BBB, the ability of ALC NP, which can reach BBB, to stimulate the production of this neurotrophin directly in the nervous system, makes acetyl L-carnitine in nanoparticle form a good candidate to protect brain from neurodegenerative diseases.

In conclusion, considering that low amount of secreted S100B is considered neurotrophic and that the NGF secretion promotes cell plasticity and neurite outgrowth, our results, which show that ALC NPs decrease the secretion of S100B and increase the production of NGF is a very encouraging finding, suggesting that, in the future, a formulation of ALC NP could represent a good strategy versus the nervous system senescence. The present study has some limitations. From the nanoparticle production point of view, future investigations will be devoted to the evaluation of in vitro release kinetics of ALC from the milled nanosystems in simulated physiological fluids, which represents a crucial step to fully understand their biopharmaceutical behavior. From the biological point of view, the limitation is due to the use of an in vitro cell model, based on undifferentiated SH-SY5Y neuroblastoma cell line, which is not a direct model of neuroinflammation, as stated above for the S100B release. We are planning to set up a further in vitro cell model using primary neurons, as well as an in vitro BBB model to fully recapitulate CNS microenvironment. Moreover, studies aimed to clarify the mechanism of entrance of ALC NP in cells will be performed to elucidate the intracellular ALC NP concentration compared with ALC in bulk form and therefore its bioavailability. Finally, preclinical studies in animals will be necessary to confirm the effects of this formulation in various neurodegenerative disease models and to further assess the BBB penetration capacity, biosafety and efficacy.

## 4. Materials and Methods

### 4.1. ALC Nanoparticle (NP) Preparation

Acetyl L-carnitine (ALC) raw powder in bulk formulation for human use was obtained from ACEF SpA (Fiorenzuola D’Arda, PC, Italy) and its granulometry was determined and validated by certified sieves (Filtra Vibraciòn—Barcelona- ES). Nanoparticles were prepared in a planetary ball mill in the AMBIOTEC lab (Cisterna di Latina (LT), Italy). To optimize nanoparticle synthesis, many tests were performed in which the powder/balls ratio, the size of the balls, the time and the speed of milling were varied. The best conditions were obtained by mixing 98% acetyl L-carnitine powder (20 g) with 2% E1201 additive (polyvinylpyrrolidone, PVP—CAS number: 9003-39-8, 0.4 g) certified for human use. The process was performed using 10 g of zirconium balls (2 mm diameter) in zirconium jars. The mixture was solubilized 1:3 (*w*/*v*) in ultrapure water and milled in a planetary ball mill for seven hours at 700 rpm. The wet mixture was recovered, dried in a vacuum oven at 105 °C for 72 h and stored at room temperature for further analyses and experiments.

### 4.2. Physical Characterization of Nanoparticles

The dry ALC NP powder was solubilized in ultrapure water at a final concentration of 1 mg/mL and subsequently filtered on a 0.45 μm cellulose acetate membrane. The dispersion was thoroughly mixed until complete solubilization. Samples from three independent preparations were analyzed by Dynamic Light Scattering (DLS) using a Zetasizer Nano apparatus (Malvern Instruments Ltd.) equipped with a 4 mW HeNe laser source (632.8 nm). All measurements were carried out in triplicate at 25 °C. To evaluate the stability, a suspension of ALC NP was prepared and analyzed over a period of one month by measuring the size of the particles over time (freshly prepared, after 1, 2, 7, 14 and 30 days). In the interval between one measurement and another, the solution was stored at +4 °C, and the DLS analyses were always performed on the same suspension.

To determine the shape, primary size, size distribution, and agglomeration status, ALC NP was characterized by electron microscopy using scanning electron microscopy (SEM) (FE-SEM Auriga, Carl Zeiss Microscopy GmbH, Jena, Germany) equipped with a Soft Imaging System. Samples were prepared for the analysis as previously described [18].

### 4.3. Thermogravimetric Analysis (TGA)

Thermogravimetric analysis was carried out by using a Mettler TG50 thermobalance equipped with a Mettler TC 10 A processor (Mettler Toledo, OH, USA). All measurements were carried out under nitrogen flow (25 mL/min) by heating 5–10 mg of sample in a 25–600 °C temperature range at 10 °C/min.

### 4.4. Cell Culture

The human neuroblastoma cell line SH-SY5Y was obtained from the American Type Culture Collection (ATCC; CRL-2266, Rockville, MD, USA). The SH-SY5Y cells were cultured in a humidified atmosphere containing 5% CO_2_ at 37 °C until they reached approximately 80% confluence, as determined using an inverted optical microscope. Cells were maintained in Dulbecco’s Modified Eagle Medium (DMEM F12 Medium) (Gibco, Thermo Fisher Scientific, Waltham, MA, USA) supplemented with 1% L-glutamine, 1% penicillin/streptomycin (Sigma-Aldrich, Co. Saint Louis, MO, USA), and 10% Fetal Bovine Serum (FBS) (Sigma-Aldrich).

### 4.5. Cell Treatment

Cells were left untreated (CTL) or stimulated with 10 ng/mL TNF-α and treated for the required time, according to each experimental condition, with different concentrations of ALC and ALC NP, both in the presence and absence of TNF-α stimulation. ALC and ALC NP suspensions were freshly prepared before each experiment. Aside from cell viability experiments, all the others were performed using the two lowest and intermediate non-cytotoxic concentrations, 0.01 mM and 0.1 mM, and using always employing freshly prepared solutions containing nanoparticles of 142 nm in size, in order to maintain a focus on dose minimization, in line with potential therapeutic translation strategies. PVP alone was tested in the same concentration used in ALC NP formulations (mass ratio ALC:PVP = 50:1).

### 4.6. MTS Assay

To evaluate the cytotoxic effects of different concentrations of ALC and ALC NP on SH-SY5Y cell line, a colorimetric assay based on MTS (3-(4,5-dimethylthiazol-2-yl)-5-(3-carboxymethoxyphenyl)-2-(4-sulfophenyl)-2H-tetrazolium) was performed. In summary, SH-SY5Y cells were seeded at a density of 5 × 10^3^ cell per well in 96-well plates. The following day, cells were either left untreated (control, CTL) or exposed to various concentrations of ALC and ALC NP for 24, 48, 72 h, and 7 days. At each time point, 100 µL of MTS reagent was added to each well. After a 3 h incubation period, absorbance was measured directly at 492 nm using a spectrophotometer.

### 4.7. RNA Extraction and Reverse Transcription

The SH-SY5Y cell line was either left untreated (CTL), stimulated with 10 ng/mL TNF-α for 30 min, or pre-treated for 1 h with 0.1 mM or 0.01 mM ALC and ALC NP before stimulation with 10 ng/mL TNF-α for 1 h. For the analysis of NGF mRNA expression, SH-SY5Y cell line was left untreated or treated with 0.1 mM or 0.01 mM ALC and ALC NP for 24 h and 48 h. Total RNA was isolated from both treated and untreated cells using the Blood/Tissues Total RNA Extraction Kit (Fisher Molecular Biology, Trevose, PA, USA) and subsequently reverse-transcribed into cDNA using the ImProm-II reverse transcriptase (Promega Corporation, Madison, WI, USA), following the manufacturer’s protocol. The quality control of RNA was assessed using an agarose gel and spectrophotometric measurements, verifying the 260/280 ratio.

### 4.8. Quantitative Real-Time PCR

Quantitative real-time PCR (qRT-PCR) was performed using the ABI Prism 7300 system (Applied Biosystems, Thermo Fisher Scientific, Waltham, MA, USA). Amplification reactions were carried out with the Sensimix Plus SYBR Master Mix. Primers (listed in Table 2) were synthesized by Bio-Fab Research and designed using Primer Express software version 1.4.0 (Applied Biosystems). Gene expression levels were calculated using the 2^−ΔΔCt^ method, normalized to the 18S rRNA gene as an internal control.

### 4.9. Immunofluorescence Analysis

Immunofluorescence analysis was used to detect total p65 and phosphorylated p65 (p-p65) proteins. SH-SY5Y cells were seeded at a density of 5 × 10^3^ cells/cm^2^. The following day, cells were either left untreated (CTL), stimulated with 10 ng/mL TNF-α for 10 min, or pre-treated for 1 h with 0.1 mM or 0.01 mM ALC and ALC NP prior to stimulation with 10 ng/mL TNF-α for 10 min. Cells were then fixed with ethanol for 15 min at room temperature (RT) and permeabilized with 0.5% Triton X-100 in PBS for 10 min at RT. To block non-specific binding, cells were incubated with 3% bovine serum albumin (BSA) in PBS for 30 min. Subsequently, cells were incubated for 1 h at RT with primary antibodies against total p65 and phospho-p65 (Abcam, Cambridge, UK) (1:200 dilution). After washing with PBS, cells were incubated with Alexa Fluor 595-conjugated donkey anti-rabbit secondary antibody (Invitrogen, Thermo Fisher Scientific, Waltham, MA, USA) (1:400 dilution) for 1 h. Nuclei were counterstained with DAPI. All steps were carried out at room temperature. Images were acquired using a Leica DM IL LED microscope equipped with an AF6000 modular imaging system (Leica Microsystems, Milan, Italy).

### 4.10. ELISA

SH-SY5Y cells were seeded at a density of 1 × 10^5^ cells/cm^2^. The following day, cells were either left untreated (CTL), stimulated with 10 ng/mL TNF-α for 1 h, or pre-treated with 0.1 mM or 0.01 mM ALC and ALC NP for 1 h prior to stimulation with 10 ng/mL TNF-α for an additional hour. The concentrations of IL-6, IL-8, and IL-1β in the supernatants from both treated and untreated SH-SY5Y cells were quantified using ELISA kits (Fine Test ELISA, Fine Biotech Co., Ltd., Wuhan, China), following the manufacturer’s protocols. Moreover, the day after seeding, cells were either left untreated (CTL) or treated with 0.1 mM or 0.01 mM ALC and ALC NP for 48 h and 72 h to determine the concentrations of NGF/NGFβ in the supernatants, or for 24 h to determine that of S100B, from both treated and untreated SH-SY5Y cells. The concentrations were quantified using ELISA kits (Fine Test ELISA, Fine Biotech Co., Ltd., Wuhan, China), following the manufacturer’s protocols. Absorbance was measured at 450 nm using a microplate reader (NeBiotech, Holden, MA, USA).

### 4.11. Calcium Assay

The intracellular Ca^++^ was measured using Calcium Green-1 AM (Invitrogen, Thermo Fisher Scientific) following the manufacturer’s instructions, using cells seeded at a density of 5 × 10^4^/cm^2^ and treated with 0.1 mM ALC and ALC NP for 5 min, 20 min, and 1 h. The assay is based on the use of molecules that exhibit an increase in fluorescence upon binding Ca^++^.

### 4.12. Densitometric Analysis

The open-source ImageJ software v1.54p (https://imagej.nih.gov/ij/, accessed on 1 July 2024) was used to perform densitometric analysis of protein expression. For each cell culture condition, the integrated fluorescence density values obtained in immunofluorescence experiments were considered. To calculate p65 pixel intensity and to quantify nuclear translocation, we applied the following formula: *N/C = Fnucl/Fcyt*, which takes into account the ratio between nuclear fluorescence and cytoplasmic fluorescence.

### 4.13. Statistical Analysis

Statistical analysis was performed using Prism 5.0 software (GraphPad Software, San Diego, CA, USA). Data distribution was evaluated for deviations from normality, and then two-way repeated measure ANOVA followed by Bonferroni post hoc tests were applied to evaluate differences between groups, whereas experiments with a single-factor design were analyzed using one-way ANOVA followed by a multiple-comparison post hoc test. A *p*-value of less than 0.05 was considered statistically significant. All data were collected from a minimum of three independent experiments, with each experiment conducted in duplicate or triplicate.

## Figures and Tables

**Table 1:** Hydrodynamic diameter and polydispersity of ALC NP during time (from 0 to 30 days).

Sample	Hydrodynamic Diameter (nm)	PDI
ALC NP_0	142 ± 1	0.19 ± 0.01
ALC NP_1	176 ± 4	0.26 ± 0.03
ALC NP_2	230 ± 2	0.23 ± 0.02
ALC NP_7	249 ± 3	0.30 ± 0.03
ALC NP_14	303 ± 19	0.30 ± 0.04
ALC NP_30	1883 ± 869	0.75 ± 0.24

**Table 2:** RT-PCR primer sequences.

Gene Accession Number	Primer Forward Primer Reverse
IL-6 NM_000600	5′-GATGGATGCTTCCAATCTG-3′ 5′-CTCTAGGTATACCTCAAACTCC-3′
IL-8 NM_000584	5′-GACATCAAAGAAGGACTTG-3′ 5′-GCCACAATTTCAGATCCTG-3′
IL-1β NM_000576	5′-ACAGAATCTCCGACCACCACTA-3′ 5′-TCCATGGCCACAACAACTGA-3′
NGF NM_002506	5′-TTCAACAGGACTCACAGGAG-3′ 5′-TGACACTGTCACACACGAG-3′
18S NM_003286	5′-CGCCGCTAGAGGTGAAATTC-3′ 5′-CATTCTTGGCAAATGCTTTCG-3′

## Data Availability

The original contributions presented in this study are included in the article/Supplementary Material. Further inquiries can be directed to the corresponding author.

## Supplementary Materials

The following supporting information can be downloaded at https://www.mdpi.com/article/10.3390/ijms27157024/s1.
