# Supplementary material for: Acetyl L-Carnitine Nanoparticles Modulate Neuronal and Inflammatory Responses in In Vitro Cell Model
Source: Int J Mol Sci. 2026 Aug 5;27(15):7024. doi: 10.3390/ijms27157024 (PMC13466921; doi:10.3390/ijms27157024)
Supplement: Supplementary file 1 [file ijms-27-07024-s001.zip › ijms-4459135-supplementary.pdf]

Supplementary materials

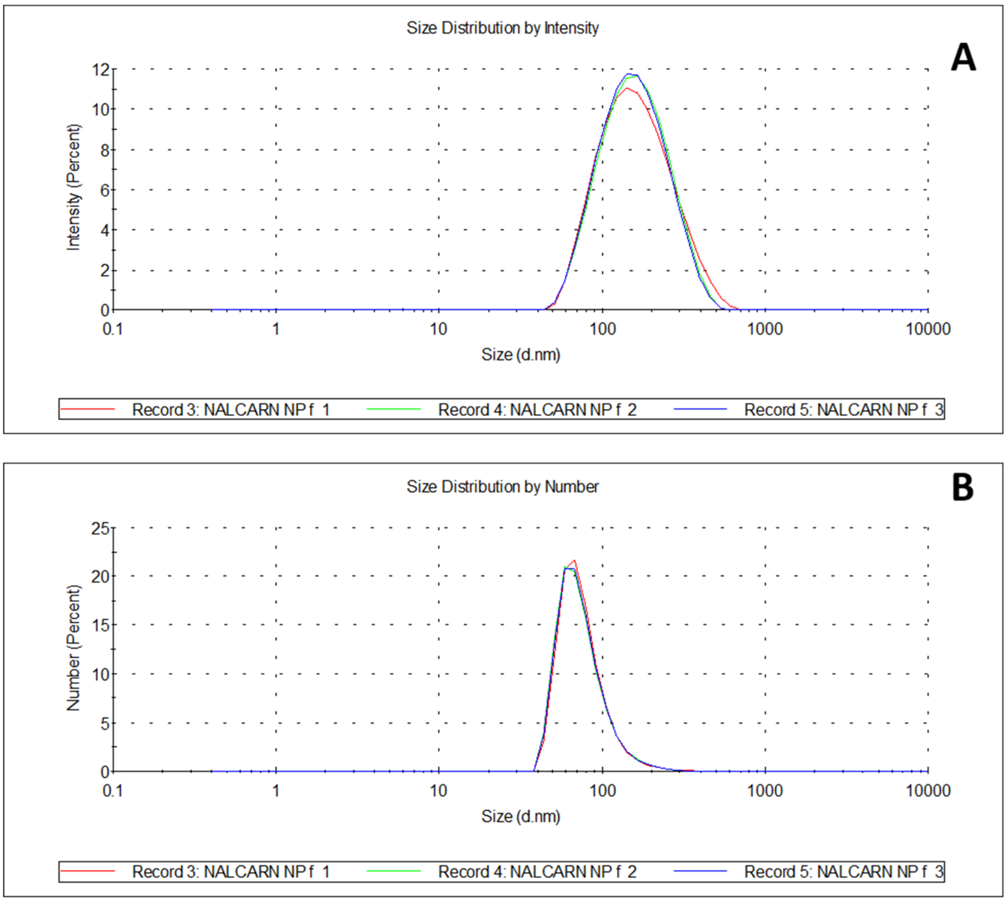

Figure S1. A) Size distribution by intensity and **B**) by number.

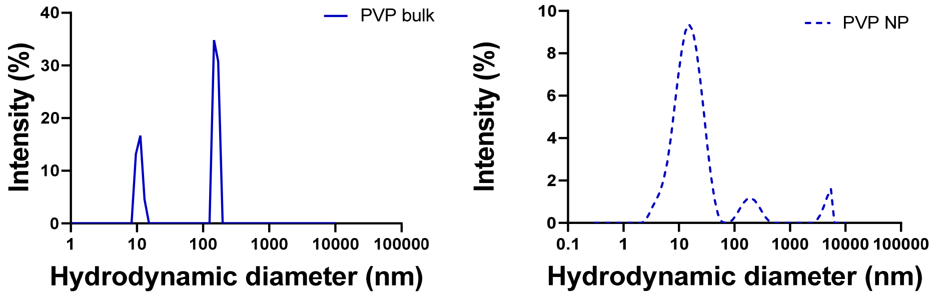

Figure S2. Size distribution of PVP bulk and PVP NP.

Table S1. Hydrodynamic diameter and PDI of PVP bulk and PVP NP.

Sample name	Hydrodynamic diameter (nm)	PDI
PVP NP	14.82 ± 0.28	0.40 ± 0.03
PVP bulk	889.3 ± 0.3	0.72 ± 0.09

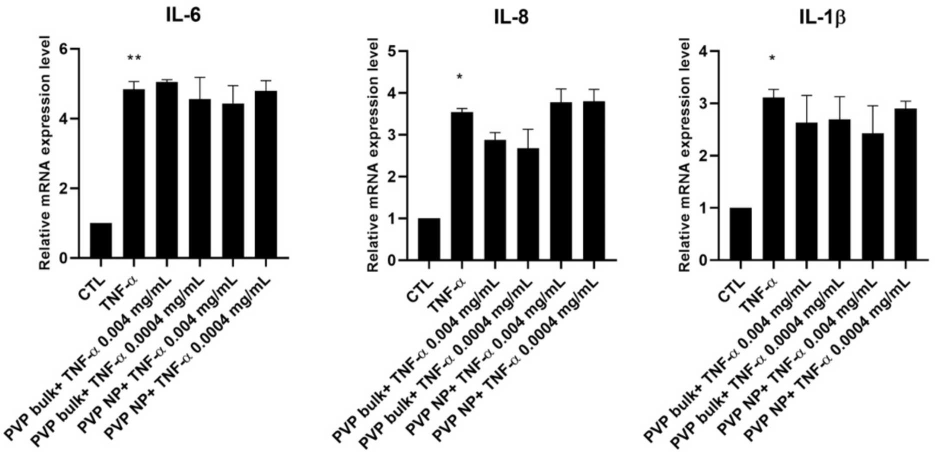

Figure S3. Effects of PVP and PVP NP on interleukins expression levels under TNF-α stimulus were analysed. Cells were left untreated (CTL), or stimulated with 10 ng/mL TNF-α for 30 min, or treated with 0.004 mg/mL and 0.0004 mg/mL concentrations of PVP or PVP NP for 1 h and then stimulated with 10 ng/mL TNF-α for 30 min. After treatments mRNA was extracted and analysed by RT-PCR. IL-6, IL-1β and IL-8 mRNA level was reported as relative mRNA expression level with respect to 18S mRNA ($2^{-\Delta\Delta Ct}$ method). * $p<0.05$ and ** $p< 0.01$ TNF- α vs. CTL

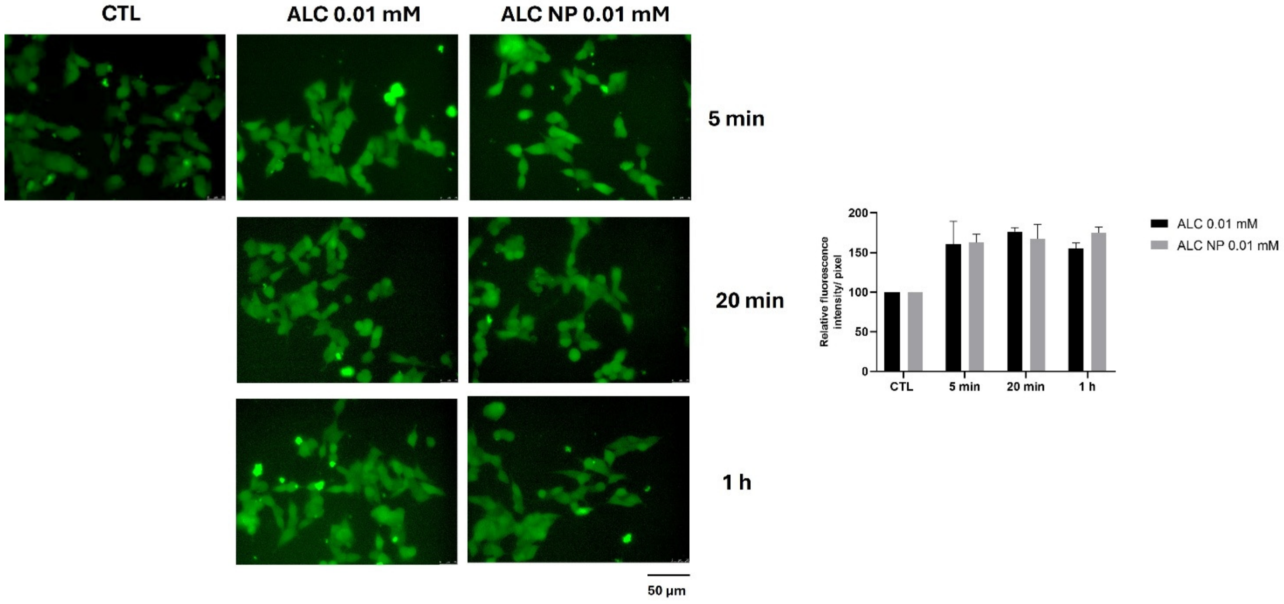

Figure S4. Effects of ALC and ALC NP on intracellular Ca^{2+} concentration. Cells were treated with 10 μM ALC or ALC NP for 5 min, 20 min and 1 h, then the amount of intracellular Ca^{2+} concentration was measured by Calcium Green-1 AM. The densitometric analysis (right) was performed by ImageJ. Results are expressed as mean ± SD of data obtained by three independent experiments.
